# The Pictures by Category and Similarity (PiCS) database: A multidimensional scaling database of 1200 images across 20 categories

**DOI:** 10.3758/s13428-025-02732-0

**Published:** 2025-06-30

**Authors:** Arryn Robbins, Michael C. Hout, Ashley Ercolino, Joseph Schmidt, Hayward J. Godwin, Justin MacDonald

**Affiliations:** 1 Department of Psychology, University of Richmond, 114 UR Drive, Rm 113, Richmond, VA 27303 USA; 2 Department of Psychology, New Mexico State University, Las Cruces, NM USA; 3 Department of Kinesiology, New Mexico State University, Las Cruces, NM USA; 4 Department of Psychology, University of Central Florida, Orlando, FL USA; 5 Department of Psychology, Southampton University, Southampton, UK

**Keywords:** Visual similarity, Multidimensional scaling, Image database, Categorization

## Abstract

Visual similarity is an essential concept in vision science, and the methods used to quantify similarity have recently expanded in the areas of human-derived ratings and computer vision methodologies. Researchers who want to manipulate similarity between images (e.g., in a visual search, categorization, or memory task) often use the aforementioned methods, which require substantial, additional data collection prior to the primary task of interest. To alleviate this problem, we have developed an openly available database that uses multidimensional scaling (MDS) to model the similarity among 1200 items spread across 20 object categories, thereby allowing researchers to utilize similarity ratings within and between categories. In this article, we document the development of this database, including (1) collecting similarity ratings using the spatial arrangement method across two sites, (2) our computational approach with MDS, and (3) validation of the MDS space by comparing SpAM-derived distances to direct similarity ratings. The database and similarity data provided between items (and across categories) will be useful to researchers wanting to manipulate or control similarity in their studies.

## Introduction

Similarity plays an important role in many aspects of visual cognition, influencing the way that we find and recognize faces and objects (Biederman, 1987; Edelman, 1998; Hebart et al., 2023), how we learn and form categories (Goldstone, 1994; Nosofsky, 1986), how we search for and remember objects (Hout & Goldinger, 2015; Guevara Pinto, Papesh, & Hout, 2020), and much more. The ability to quantify and manipulate similarity is essential for addressing research inquiries in visual cognition. For example, in visual search, researchers may want to investigate the influence of target/distractor similarity on response time, and might do so by changing feature values along one or more dimensions of a target to develop a set of similar or dissimilar distractors (Duncan & Humphreys, 1989). In category learning experiments, similarity can be manipulated by altering the feature space of the stimuli, such as changing the color or texture of objects, to assess how these variations impact learning and categorization (Bohil et al., 2023; Ercolino et al., 2020; Nosofsky, 1986). Additionally, researchers may control for (rather than manipulate) similarity in their stimuli. For instance, in studies exploring facial recognition, researchers often employ morphing techniques to systematically vary facial features while maintaining a degree of similarity, allowing for precise control over the level of resemblance between faces (Jenkins & Burton, 2011). In object recognition tasks, researchers might use a standardized set of objects that are similar in size and color but differ in shape, ensuring that similarity in non-target dimensions is controlled for (Brady et al., 2008).

In response to the need to control for and manipulate similarity, researchers have developed methodologies to quantify or model similarity among (often visual) stimuli. These methods often include human similarity judgments obtained by using Likert scales or odd-one-out tasks (Hebart et al., 2020, 2023), or computational methods like multidimensional scaling (MDS) that utilize similarity ratings (obtained in a variety of different ways; see Daggett & Hout, 2025, for a tutorial review) to model the relationships among the items (Hout et al., 2013, 2016). The process of obtaining and validating measures of similarity has been a challenge for researchers, as this can be time-consuming and require many research participants. Thus, many researchers rely on established stimuli databases to save time and resources when investigating (or utilizing) similarity in their research.

In the vision sciences, researchers frequently employ image databases which provide a set of freely available images, often organized into categories, for use in such paradigms as visual search, categorization, object identification, item memory, and more. In addition to the stimuli themselves, several of these databases provide similarity ratings between images of objects for researchers to use (Frank et al., 2020; Hebart et al., 2023; Horst & Hout, 2016; Jiang et al., 2022; Nosofsky et al., 2018). For example, the MM-MDS database (Hout et al., 2014) provides similarity ratings that have been scaled via MDS for 240 categories of objects, each with 16 or 17 exemplars sampled from the massive memory database (Brady et al., 2008).

To provide visual cognition researchers with the ability to quantify and manipulate visual similarity in stimuli, here, we build upon previous databases by developing and validating an image set of 1200 items spread across 20 categories. Our image database provides similarity ratings between objects, allowing researchers to examine both within-category and across-category similarity. With this set of stimuli, researchers can, for example, manipulate similarity compared to a prototype (or central exemplar) to see the effects of varying levels of typicality on categorization or visual search tasks, or they can examine within-category similarity (e.g., Robbins & Evdokimov, 2024) to examine how category heterogeneity influences attention or category verification decisions.

### Modeling similarity using multidimensional scaling

One popular method for modeling image similarity is MDS (Hout, Papesh, & Goldinger, 2013), which offers distinct advantages over alternative methods employed to measure the perceptual relationships between images. While various techniques such as human-derived ratings (e.g., Likert ratings of similarity without MDS) and computer vision algorithms have been employed, MDS is particularly valuable because of its capacity to extract the latent structure of similarity spaces.

Like any other modeling technique, MDS operates under certain mathematical assumptions, such as treating psychological similarity data as satisfying properties of a distance metric (e.g., symmetry) and modeling these distances within a Euclidean space. However, a key advantage of MDS, especially when compared to feature-driven algorithmic approaches, is that it does not require a priori assumptions about the *psychological* structure of the similarity space. Specifically, MDS does not impose predefined features (e.g., “color” or “shape”) or dimensions on the data. Instead, it allows the psychological organization of objects to emerge from participants’ similarity judgments themselves. By adopting this perspective, MDS transforms collected similarity data into a reduced-dimensional space, preserving the relative distances between images as perceived by participants. By placing emphasis on the participants’ subjective judgments, MDS facilitates the emergence of similarity spaces that reflect the nuances of human cognition without being confined by preconceived notions regarding the dimensions upon which similarity is judged or perceived. This property is particularly advantageous when dealing with complex visual stimuli that might elude direct mathematical representation.

Researchers across many psychological sub-disciplines have used MDS to quantify the similarity among groups of items (i.e., the item set; Jaworska & Chupetlovska-Anastasova, 2009). There are different versions of MDS, but in general, each type of MDS analysis that is used in psychological research takes in similarity information (collected from human raters) between each item and all other items in the set and uses data-reduction procedures to reduce complexity in the corresponding similarity matrix. The subsequent output of the analysis is a set of coordinates (in a multidimensional space) for each item. The coordinates can then be used to obtain distances between each item and all other items for the purposes of examining similarity relationships. MDS allows for the extraction of multiple featural dimensions and can often be used to provide a visual appreciation of the underlying relational structures that were used to govern the similarity ratings.

The first step in modeling similarity relationships is to acquire similarity ratings to be used as input in the MDS analysis. This information can come from many sources which include: subjective similarity ratings (e.g., Likert ratings) obtained using a pairwise method (i.e., participants view two images and rate them according to perceived similarity) or multiple-item methods such as the Spatial Arrangement Method (SpAM; Goldstone, 1994). During a SpAM task, many (or all) of the items from the set are presented simultaneously, and participants are instructed to arrange the items on the computer monitor so that items that they perceive to be more similar are closer together in space (and vice versa). In other words, similar items are to be placed close to one another, and dissimilar items are placed proportionately farther away. A proximity matrix is then obtained from the pairwise Euclidean distances (measured in pixels) between every pair of items. The benefit of using SpAM over other methods of collecting similarity data is that it is fast and efficient and produces output data of equal quality compared to popular (but much slower) methods like pairwise ratings (Hout et al., 2013, 2016; Richie et al., 2020).

### The PiCS database

In response to the need for an easily accessible and comprehensive image similarity resource, we developed a novel image database that used MDS to model the similarity within and between all category exemplars for a collection of 1200 items across 20 distinct object categories. We expand upon a related database, the MM-MDS, with more objects per category. We also provide users the ability to obtain both within- and between-category similarity estimates for each item, enabling comparisons across categories (e.g., “butterfly” vs. “bird”) as well as within-category (e.g., similarity among different “bird” exemplars).

The development of the database consisted of two experiments that involved collecting similarity ratings and subsequently validating those ratings. In Experiment 1, we gathered similarity ratings via SpAM tasks, acquiring data from participants at multiple sites. Our computational approach leveraged MDS to distill these ratings into a multidimensional similarity space. In order to validate the robustness of the generated MDS space, we conducted Experiment 2 to correlate raw similarity ratings for a subset of pairs with the distances in MDS space from Experiment 1.

## Experiment 1 Method

### Participants

Three hundred and thirty-four participants from two institutions (New Mexico State University and the University of Central Florida) participated in the study for course credit. Informed consent was obtained from all individual participants included in the study. Institutional Review Board approval was granted for data collection from both institutions. All had normal or corrected-to-normal vision.

### Stimuli

Stimuli were selected from the Bank of Standardized Stimuli (BOSS; Brodeur et al., 2014), the Massive Memory Database (Brady et al., 2008; Konkle et al., 2010; Hout et al., 2014), Hemera Photo Objects collection, and the Teddy Bear Encyclopedia (Cokrill, 1993). Each was resized to an area of 8000 pixels with aspect ratios free to vary. See Table 1 for an example from each category.

**Table 1:**
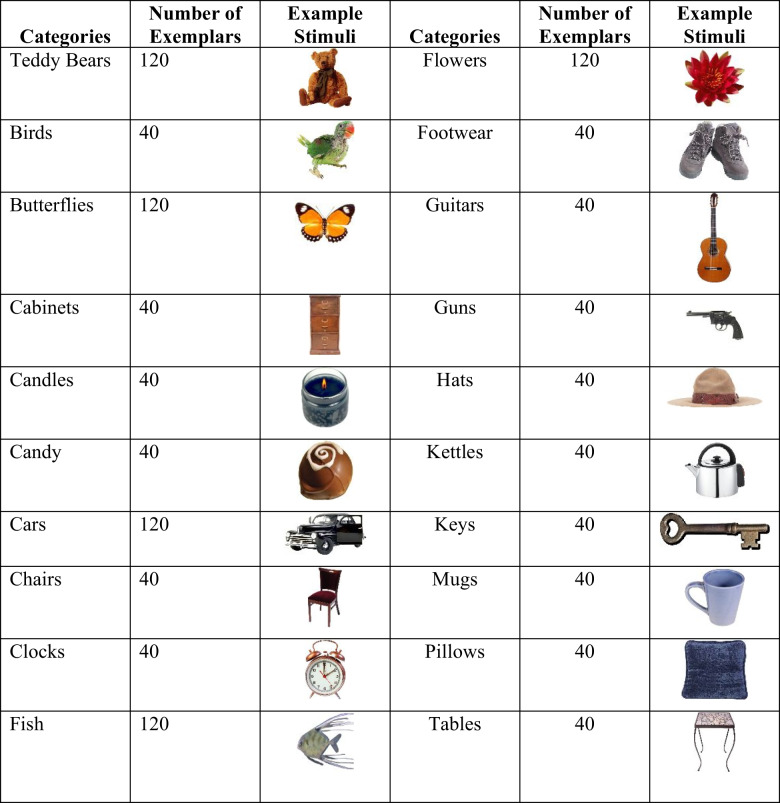
Categories used in the PiCS Database

### Apparatus

Data collection was conducted using EPrime vers. 2 (Psychology Software Tools, Pittsburgh, PA, USA) at two separate laboratories (one at New Mexico State University and one at the University of Central Florida). At each site, the stimuli were presented on an Asus PB287Q 4 k monitor with a refresh rate of 60 Hz and screen resolution of 3840 × 2160.

### Design and procedure

**Fig. 1:**
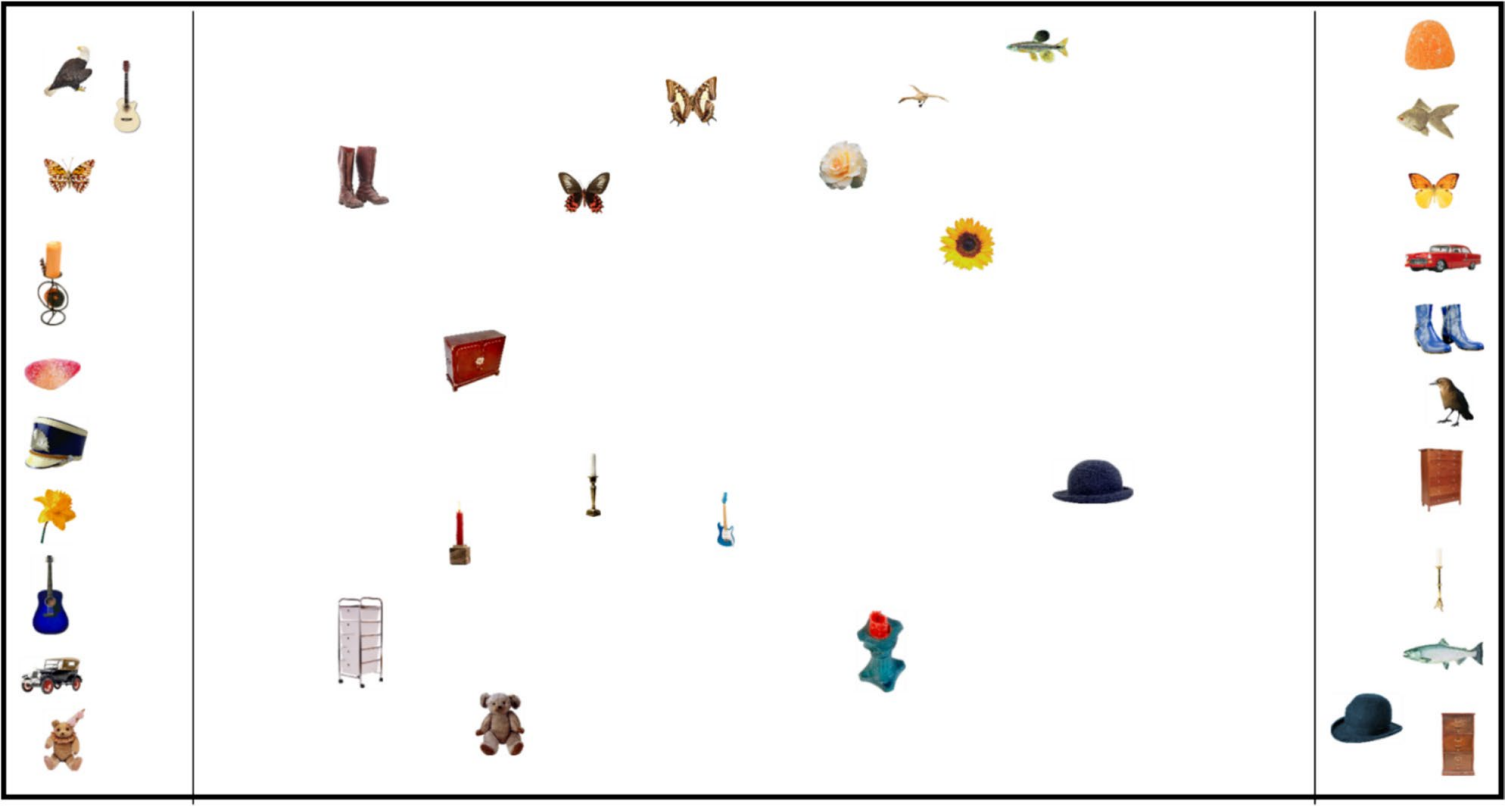
Layout of SpAM task. *Note*: During a trial, participants viewed 36 objects on the computer screen. The objects appeared on the outside edges of the main gallery. Participants were instructed to drag and drop the images into the gallery (the center of the screen) using the cursor, and arrange the images by similarity so that items closer together in space were those they perceived to be more similar. Please note that the images in this figure are not to scale, and all items in the original experiment were resized to an area of 8000 pixels with aspect ratios free to vary

We used the spatial arrangement method to collect similarity ratings as it has been shown to be a valid way of collecting similarity ratings where individual differences do not markedly contribute to the overall MDS solution (Hout et al., 2013). Participants were instructed to complete as many trials as they could in 50 min, and to focus on the accuracy rather than the speed of their similarity judgments. During a trial, participants viewed 36 objects on the computer screen. The objects appeared on the outside edges of the main gallery (see Fig. 1). Participants were instructed to drag and drop the images into the gallery using the cursor and to arrange the images by visual similarity such that items closer together in space were those they perceived to be more visually similar. It is important to note that, as with other human-subject studies, not all participants may have strictly adhered to these instructions. Participants were provided the following instructions, which requested that they focus on visual similarity:

*“When judging similarity, please try to focus on the VISUAL information that you can see rather than what the picture is; this includes the category and the function of the item. If the images look visually similar, place them close together; if they look visually dissimilar, place them farther away from each other (ignoring all other information).”*

The ordering of stimuli for the entire experiment was generated using the partial incomplete block design generation algorithm detailed in MacDonald, Hout, & Schmidt (2019). Given the number of stimuli (1200) and the number of stimuli per trial (36), the algorithm created an experimental design that ensured that each of the 719,400 pairs of stimuli were presented together in at least 3 trials. Given these requirements, the algorithm produced a design of 4563 trials, which was completed across 354 participants.

## Experiment 1 Results

### MDS algorithm

We computed a dissimilarity matrix based on the average Euclidean distance between each item and every other item (i.e., pairwise distances). This matrix is a table that shows how different or dissimilar each pair of items is from one another, and each cell in the matrix represents the dissimilarity between two items: The smaller the distance, the more similar the pair was perceived to be. These distances were then subjected to classic multidimensional scaling (MDS) using the cmdscale() function in MATLAB (MathWorks Inc., 2022). Classic MDS maps high-dimensional data onto a lower-dimensional space while linearly preserving pairwise distances. It distinguishes itself from other forms of MDS (e.g., nonmetric MDS) in that it doesn’t involve iterative stress reduction for dimensionality reduction. With a precursor to the PiCS database (the MM-MDS; Hout et al., 2014), the researchers used the INDSCAL procedure, which allowed for individual-specific multidimensional spaces. For the development of this database, we opted for classic MDS. This choice was guided by our desire to maintain a linear representation of the continuous pairwise distances measured in pixels, ensuring the scaled distances remain on a comparable scale to the original matrix, thereby simplifying interpretation. Additionally, the classic MDS approach was best for our design and a large number of stimuli, as INDSCAL would require each participant to provide a similarity rating for each stimulus pairing. 

The cmdscale function primarily requires two arguments: *d,* which is the dissimilarity matrix representing pairwise dissimilarities between items, and *k,* which specifies the number of dimensions to which the data should be reduced. For our MDS analysis, we used the dissimilarity matrix derived from the SpAM data collection and set *k* to ten dimensions (see the Dimensionality section below for our rationale behind selecting ten dimensions). The function outputs a set of coordinates in the reduced-dimensional space, where the Euclidean distances between these coordinates aim to closely match the original dissimilarities from the input matrix. Using these coordinates across the ten dimensions, we recalculated the distances between each item and all others.

### Dimensionality

**Fig. 2:**
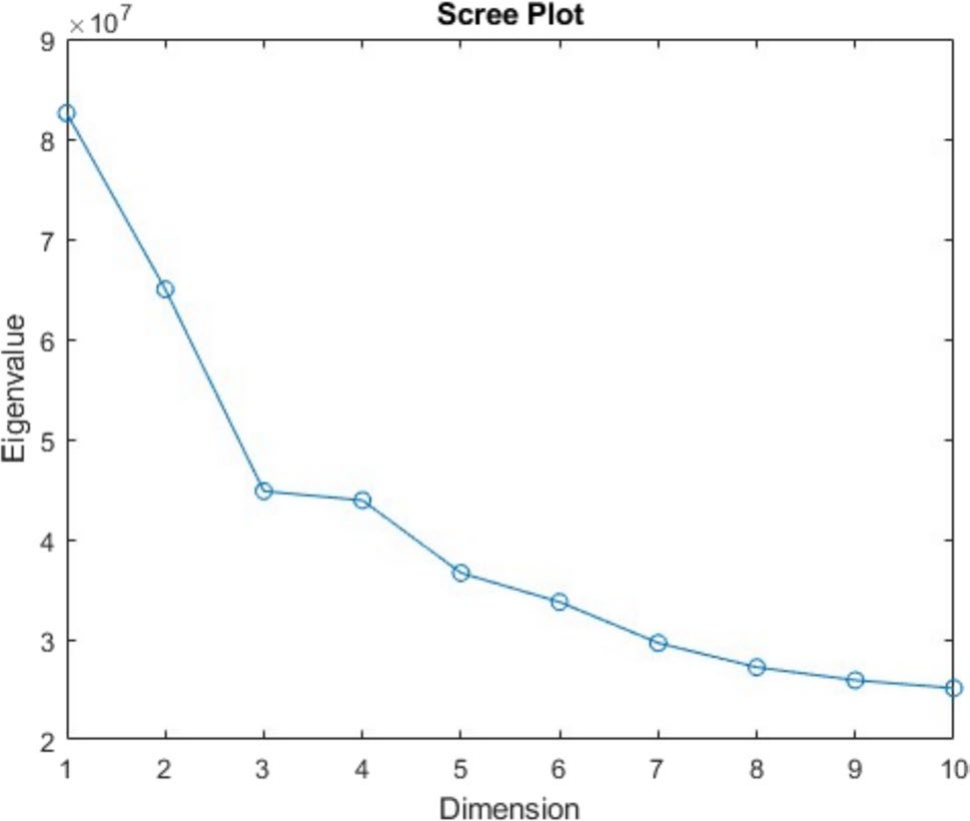
Scree plot with eigenvalues by dimensions. *Note*: Scree plot showing eigenvalues across dimensions in the PiCS database’s MDS solution. Higher eigenvalues in the initial dimensions indicate that these dimensions account for a substantial portion of the variance in perceptual similarity among items

Across different forms of MDS (e.g., classic MDS, INDSCAL, non-metric MDS), the researcher must specify the number of dimensions, k, or explore different values of k to determine the most appropriate dimensionality. In all cases, the decision involves balancing model fit, model complexity, and interpretability of the resulting dimensions. For instance, researchers may examine scree plots of eigenvalues (in classic MDS) or stress loss functions (in non-metric MDS) to guide this decision. In classic MDS, as implemented with the cmdscale function in MATLAB, the solution is derived from the singular value decomposition of the double-centered dissimilarity matrix, and the researcher can inspect how much variance is accounted for across different k values. For our analysis, we specified ten dimensions, which we felt would be a sufficient number of dimensions to capture the high dimensionality of photorealistic images and the nuances within each category without overfitting. The scree plot in Fig. 2 shows eigenvalues across dimensions in the MDS solution. Higher eigenvalues in the initial dimensions indicate that these dimensions account for a substantial portion of the variance in similarity among items. Although the scree plot suggested an ‘elbow’ at four dimensions, our choice of ten dimensions was further supported by our behavioral validation (see Fig. 2), and our prior work, which suggests that overestimating dimensionality is a more optimal approach than underestimating it (Hout et al., 2018).

## Experiment 2

Validation of our similarity data is a critical step in ensuring its reliability and applicability to visual cognition research. By establishing a relationship between perceived similarities as measured through two different behavioral methods (i.e., spatial arrangement in Experiment 1 and pairwise similarity ratings in Experiment 2), we sought to assess the robustness of the derived MDS distances. Specifically, validation here refers to examining the extent to which distances obtained from participants’ spatial arrangements correspond to independently collected direct similarity ratings. This cross-method validation allows us to test whether the dimensions identified through MDS reflect meaningful perceptual differences beyond the specific structure of the original task. This validation also establishes a basis for cross-study comparisons, enabling researchers to align their findings with a validated similarity space. For Experiment 2, we used a similarity rating task which aligns closely with the fundamental aspects of visual similarity assessment. In this experiment, participants were presented with a subset of pairs of images from our dataset and were asked to rate the similarity between each pair using a Likert scale.

### Method

#### Participants

Forty-nine participants from the University of Richmond participated in the study for course credit. All had normal or corrected-to-normal vision. Informed consent was obtained from all individual participants included in the study.

#### Stimuli and apparatus

Participants were tested in a well-lit testing room that contained four computers, allowing for up to four participants to be tested at a time. Each testing computer was a Lenovo Thinkcentre Tiny with a 22-inch LCD monitor with 1920 × 1080 resolution and a refresh rate of 60 Hz. Participants were seated approximately 24 inches from the monitor. The study ran on Eprime 3.0 (Psychology Software Tools, 2016).

#### Design and procedure

**Fig. 3:**
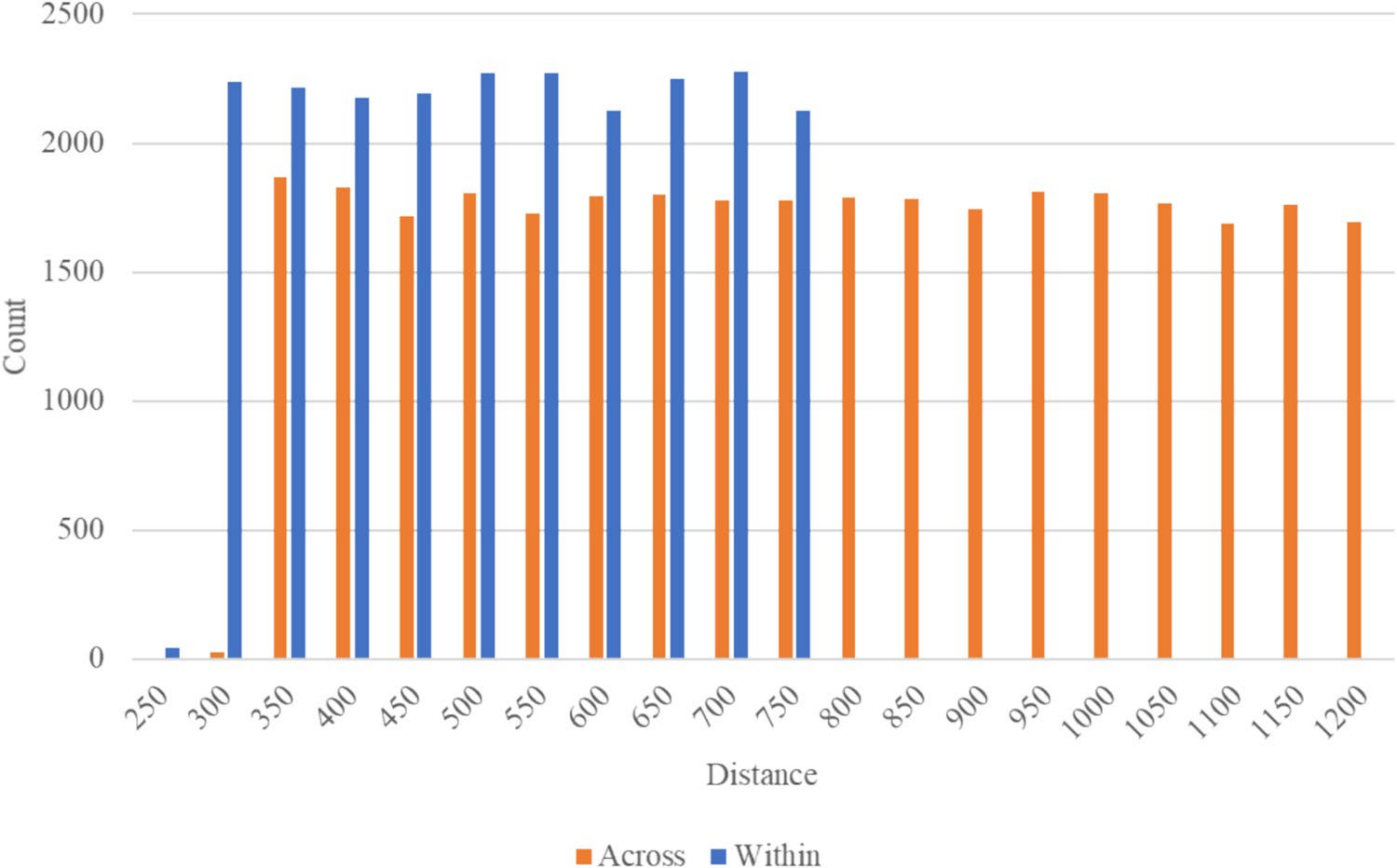
Histogram of MDS distance presentations across participants

Our primary manipulation concerned the presentation of stimuli; that is, whether pairs of images to be rated were selected from the same category (within-category trials) or were from different categories (across-category trials). The selection of image pairs from the database was such that pairs would be selected equally from across various “tiers” of similarity. For the within-category pairs, there were 29,000 possible pairs of stimuli, with 2900 pairs per tier. For the across-category pairs, there were 30,600 possible pairs of stimuli, with 1700 pairs per tier. This allowed us to select from across the range of highly similar to highly dissimilar pairs without oversampling from any part of the distribution (for either grouping of within- or across-category trials; see Fig. 3).

**Fig. 4:**
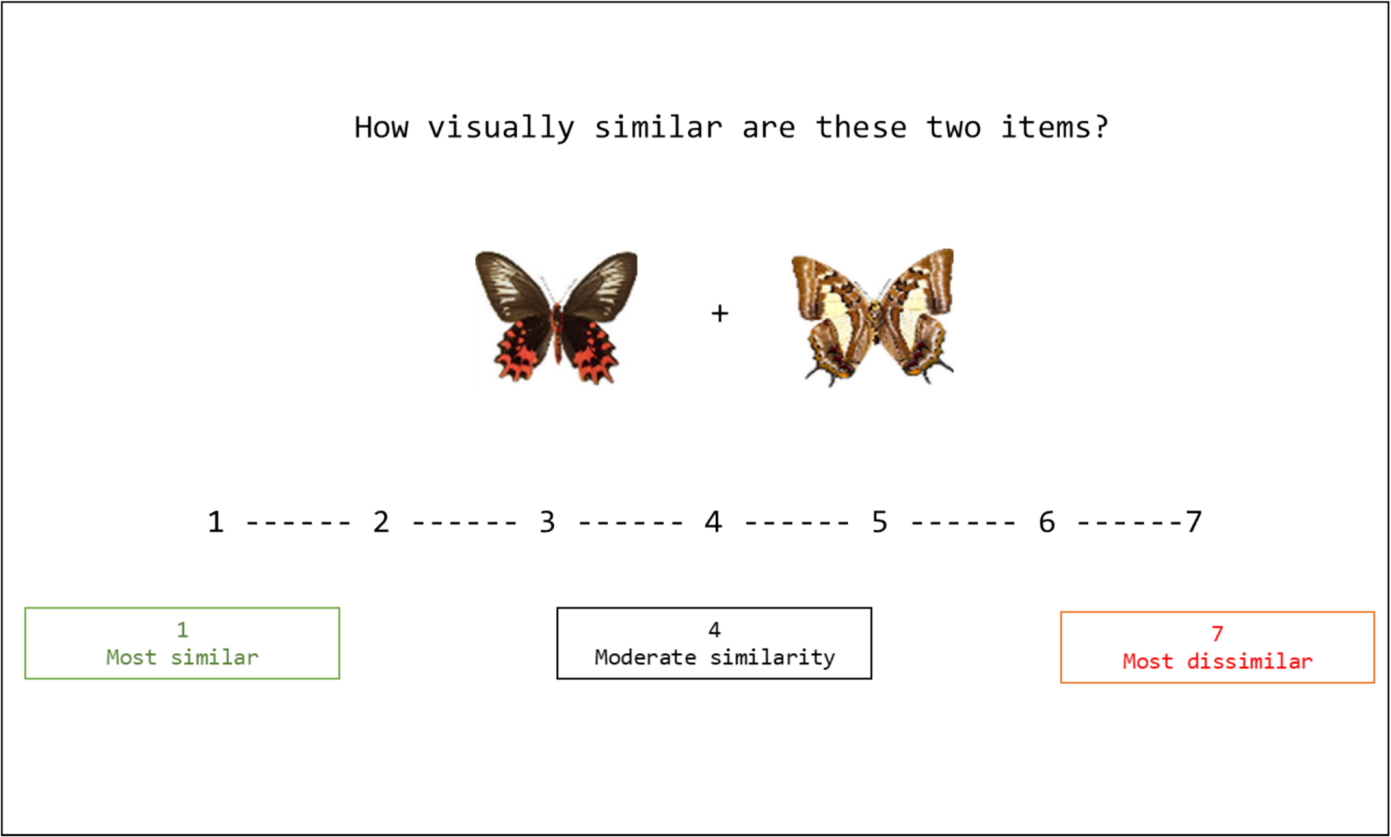
Example trial from the similarity rating task

After providing consent, participants completed the Ishihara test for colorblindness (Ishihara, 1987) and all participants had normal color vision. Once seated, participants began the study and completed trials at their own pace. Participants were instructed to complete as many trials as they could during a block. There was a total of two blocks, each lasting 20 min, with a short break between blocks. One block contained all within-category trials, and the other block was all across-category trials. The blocks were counterbalanced so that half of the participants received the within-category trials first. During a trial, participants viewed two images and were asked to provide a rating of visual similarity on a scale of 1 (highly similar) to 7 (highly dissimilar; see Fig. 4). Participants responded using the numbered row on a QWERTY keyboard. The average number of trials completed per across-category block was 663 trials (SD = 393.42) and 463 trials (SD = 224.86) for within-category blocks.

### Results

**Fig. 5:**
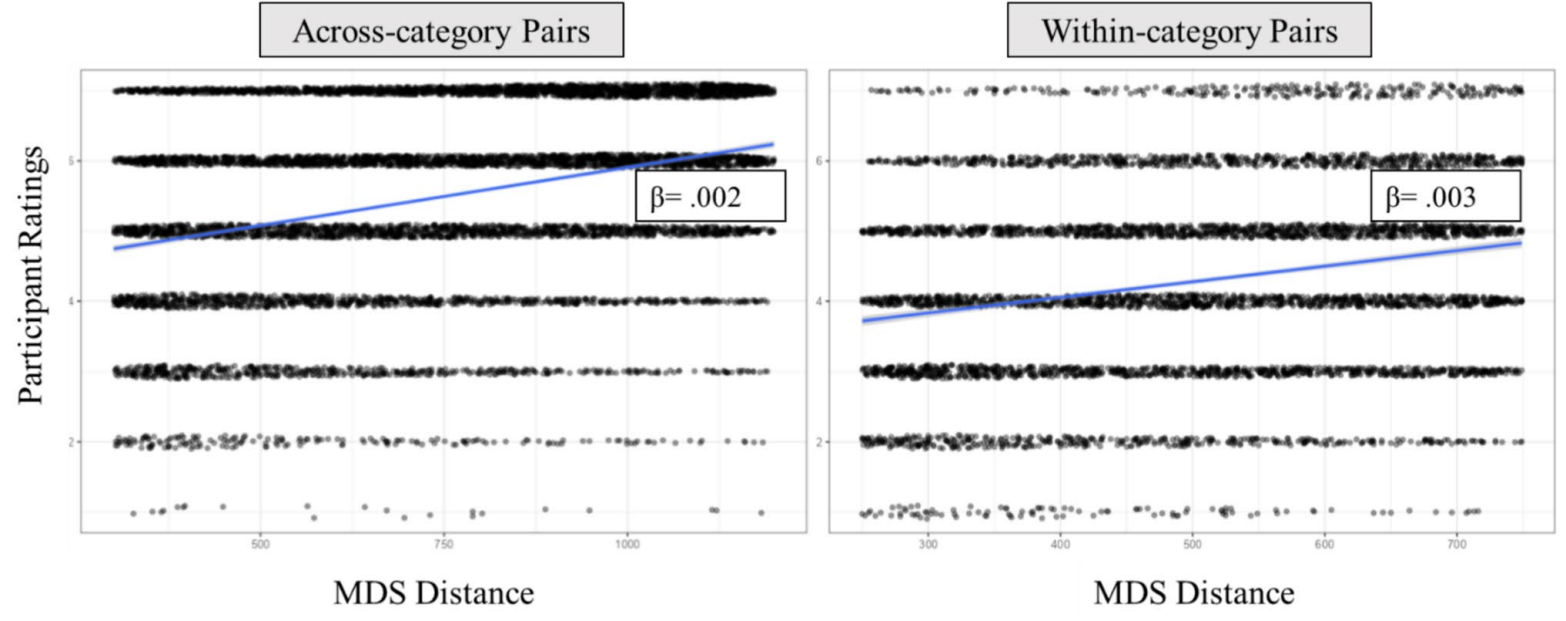
Results of linear mixed model analyses

One participant’s data was filtered from analyses because they only selected one number for all trials. We ran a confirmatory linear mixed model with MDS distance for each block type (across- or within-category), with the MDS distance as fixed effect, subject number as the clustering variable, and subject intercept as a random effect. We entered the rating for each trial as the dependent variable. For the across-category model, the *R*^2^ for fixed effects was 0.098 and 0.328 for the overall model, and for the within-category model, the *R*^2^ for fixed effects was 0.05 and 0.307 for the overall model. All effects were significant (*p* < 0.001; See Table 2 for parameter estimates and Fig. 5). As can be seen in Table 2, as MDS distance increased, so did the ratings given by participants for both across- and within-category estimates. This is what would be expected, because increasing the MDS distance was associated with participants rating the objects as more dissimilar. In sum, the results of these validation analyses indicate that the MDS distances in the PiCS database capture raw human similarity judgments and predict similarity estimates from human raters. The positive correlation here suggests that our MDS space and similarity ratings were well aligned with one another, such that objects further apart in MDS space were also rated as more dissimilar by participants in our validation experiment.

**Table 2:** Parameters and results from the linear mixed model analyses

Across-category model			Within-category model		
*Effect*	*B*	*SE B*	*Effect*	*B*	*SE B*
*Intercept*	5.48*	.16	*Intercept*	4.41*	.180
*Distance*	.002*	4.31e-5	*Distance*	.003*	1.03e-4
*R*^*2*^ =.098			*R*^*2*^ =.05		

^*^ p <.001

In order to ensure the PiCS database adequately captures the nuanced patterns of similarity judgments within categories, we analyzed within-category correlations between MDS distances and similarity ratings separately. We found some variability in correlation strengths between categories. For example, the correlation for birds was *r* = 0.04, whereas candy yielded a stronger correlation of *r* = 0.40 (see Table 3 for a complete list of correlations). By including these correlations, we provide PiCS users the ability to identify categories aligned with their research goals, reinforcing the database’s flexibility and adaptability across studies.

**Table 3:** Correlations between SpAM-derived distances and within-category ratings for each category

Category	Correlation (*r*)
Bird	.04
Guitar	.08
Shoes	.10
Gun	.13
Hat	.15
Kettle	.16
Fish	.16
Mug	.17
Pillow	.17
Car	.19
Key	.21
Butterfly	.22
Chair	.23
Table	.24
Teddy bear	.26
Candle	.27
Flower	.27
Clock	.31
Cabinet	.32
Candy	.40

### General discussion

The primary objective of this project was to develop a comprehensive image database that also includes a model of perceptual similarity between a diverse collection of visual stimuli, including 1200 images of objects spanning 20 distinct categories. Our database serves as a resource available to researchers who wish to examine image similarity within the context of psychological and vision sciences. By merging human-derived similarity data, employing multidimensional scaling (MDS), and validating the MDS distances by comparing them to direct pairwise similarity ratings collected in an independent sample, we provide a robust framework that researchers can use to explore, investigate, or control for similarity in studies of visual perception and cognition. Each item within the database has coordinates for its position within a multidimensional space, allowing for MDS distance measurement to every other item. The MDS distances allow researchers to manipulate similarity both within and across distinct categories, enabling nuanced investigations into the perceptual dimensions that govern image relationships.

The results of our validation experiment, along with the MDS analysis, demonstrate PiCS database’s alignment with the demonstrated research findings. For example, Hout et al. (2016) have underscored that MDS databases characterized by a substantial number of items maintain fidelity in preserving the validity of similarity estimates. PiCS database’s large item count and the corroborative insights from prior research in developing MDS databases (Hout et al., 2014; Jiang et al., 2022) reinforces the PiCS database’s robustness and reliability, positioning it as a tool for researchers examining similarity perception with precision and depth. Finally, another hallmark of our database is that we have validated the MDS space with subsequent similarity data. This validation lends credibility, reliability, and broader applicability to the derived similarity estimates, enhancing the robustness of conclusions drawn from the database’s utilization.

### Limitations and future directions

Although the PiCS database provides a comprehensive resource for examining visual similarity, it is important to acknowledge the limitations, particularly regarding the fidelity of within-category MDS distances. First, while our validation study (Experiment 2) showed a positive relationship between MDS-derived distances and independent similarity ratings, the strength of the correlations, especially within individual categories, was modest (median *r* ≈ 0.20). We recognize that this level of correspondence is lower than ideal for researchers seeking highly precise predictions of psychological similarity within a category.

There are several possible reasons for this modest fidelity. First, while the SpAM task and direct rating tasks both involve similarity judgments, they differ in structure (simultaneous spatial arrangement vs. sequential pairwise rating), which may introduce noise when comparing across methods. Second, contextual differences between the two data collection methods may affect the fidelity. In Experiment 1, participants judged similarity across both within- and between-category items simultaneously. It is possible that the broader context influenced how participants perceived and arranged items, reducing the precision of within-category similarity measurements. Another explanation lies in the categories themselves. Some categories may naturally exhibit low perceptual variability, making it difficult for any model that includes human judgments to reliably distinguish fine-grained within-category similarities.

Given these considerations, we recommend that researchers interpret within-category MDS distances with caution, especially if their work depends on fine-grained similarity differences within a single category. The current database may be best suited for manipulations involving across-category similarity (where distances are larger and more reliable). Alternatively, it may be particularly useful when researchers are interested in general investigations of coarse-level similarity patterns rather than detailed, item-by-item precision within categories. We view these limitations as intrinsic to any similarity database built using a wide and varied stimulus set. Psychological similarity judgments are context-dependent (e.g., Goldstone et al., 1997), and perceptual spaces are likely constructed dynamically based on the stimuli presented. As such, the PiCS database is designed for researchers to either use directly or adapt to their specific experimental contexts.

There is, however, notable potential to further explore the dimensional structure of the PiCS database. Future research could further refine the within-category similarity measures, for instance, by gathering more focused within-category SpAM data or combining SpAM with additional targeted rating tasks. Additionally, while this initial project focused on estimating broad similarities across a set of categories, future research could examine interpretable psychological dimensions within the MDS solution. For instance, collecting additional data in which participants provide ratings specific to individual dimensions – such as rating image similarity based on color – would enable researchers to select stimuli based on specific dimensions of similarity, rather than the current cross-dimensional approach used in the database. In addition, analyses of smaller, homogeneous subsets of categories could be conducted to identify shared perceptual dimensions, potentially correlating these dimensions with external visual or semantic attributes such as color, texture, shape, etc. By identifying dimensions that reflect consistent perceptual or cognitive constructs, researchers could gain additional insights into the characteristics driving similarity judgments, enhancing both the interpretability and utility of the database for targeted experimental applications.

Furthermore, given the variability in correlation strength observed across categories, future research could also explore whether different levels of dimensionality yield stronger alignment between MDS-derived distances and human similarity ratings within certain categories. For instance, collecting additional similarity data using alternative dimensional structures (e.g., fewer or greater than the ten we identified) may reveal dimensionalities better suited to particular categories, thereby addressing variability and improving alignment in within-category similarity judgments. This line of inquiry could offer researchers the flexibility to select optimal dimensional models that cater to specific categories or experimental needs, providing even greater precision in investigating perceptual or conceptual dimensions within PiCS.

### Conclusion

In conclusion, our database represents a significant contribution to the field of psychological and vision sciences by offering a resource that captures the complexity of image similarity. By combining human-derived ratings, MDS, and subsequent validation, our database can facilitate research in visual cognition and other domains where image similarity plays a pivotal role.

## Data Availability

Database images, corresponding MDS coordinates and distances, and validation experiment data are available at https://osf.io/yvb8h
